# Helium/Argon-Generated Cold Atmospheric Plasma Facilitates Cutaneous Wound Healing

**DOI:** 10.3389/fbioe.2020.00683

**Published:** 2020-06-30

**Authors:** Bih-Show Lou, Jang-Hsing Hsieh, Chun-Ming Chen, Chun-Wei Hou, Hui-Yu Wu, Pang-Yun Chou, Chih-Ho Lai, Jyh-Wei Lee

**Affiliations:** ^1^Chemistry Division, Center for General Education, Chang Gung University, Taoyuan, Taiwan; ^2^Department of Nuclear Medicine and Molecular Imaging Center, Chang Gung Memorial Hospital, Taoyuan, Taiwan; ^3^Center for Plasma and Thin Film Technologies, Ming Chi University of Technology, New Taipei, Taiwan; ^4^Department of Materials Engineering, Ming Chi University of Technology, New Taipei, Taiwan; ^5^Department of Microbiology and Immunology, College of Medicine, Graduate Institute of Biomedical Sciences, Chang Gung University, Taoyuan, Taiwan; ^6^Plastic and Reconstructive Surgery and Craniofacial Research Center, Chang Gung Memorial Hospital, Taoyuan, Taiwan; ^7^Department of Pediatrics, Molecular Infectious Disease Research Center, Chang Gung Memorial Hospital, Linkou, Taiwan; ^8^Department of Medical Research, School of Medicine, China Medical University and Hospital, Taichung, Taiwan; ^9^Department of Nursing, Asia University, Taichung, Taiwan; ^10^Department of Mechanical Engineering, Chang Gung University, Taoyuan, Taiwan

**Keywords:** cold atmospheric plasma jet, wound healing, keratinocyte, cell proliferation, cell cycle

## Abstract

Cold atmospheric plasma jet (CAPJ) or non-thermal plasma jet has been employed in various biomedical applications based on their functions in bactericidal activity and wound healing. However, the effect of CAPJ generated by a particular composition of gases on wound closure and the underlying mechanisms that regulate wound healing signals remain elusive. In the present study, we investigated the impact of helium (He)- or a gas mixture of He and argon (He/Ar)-generated CAPJ on cell proliferation, which is a pivotal step during the wound healing process. With careful treatment duration control, He/Ar-CAPJ effectively induced keratinocyte proliferation and migration mediated through the activation of epithelial-to-mesenchymal transition (EMT) and cell cycle progression, which was evidenced by a decrease in E-cadherin levels and increases in N-cadherin, cyclin D1, Ki-67, Cdk2, and p-ERK levels. Rat wound healing studies showed that He/Ar-CAPJ treatment facilitated granulation tissue formation and mitigated inflammation in cutaneous tissue, resulting in accelerated wound closure. These findings highlight the possibility that He/Ar-CAPJ can be developed as a therapeutic agent for enhancing wound healing.

## Introduction

Cold atmospheric plasma jet (CAPJ), also known as non-thermal plasma jet, is mainly operated at near human body temperature (<40°C) and provides several reactive species, including electrons, ions, neutral particles, UV radiation, heat, and reactive oxygen and nitrogen species (RONS) (Isbary et al., 2013; von Woedtke et al., 2013; Weltmann and von Woedtke, 2017). CAPJ with unique constituents and characteristics has become widely used in clinical applications, such as cancer remission, control of drug resistant bacteria, root canal treatment, and promotion of wound healing (Ehlbeck et al., 2008; Daeschlein et al., 2015).

The RONS produced by CAPJ play a key role in anti-bacterial activity, which can treat infectious diseases and promote the healing effects of skin lesions (Dunnill et al., 2017). Since CAPJ possesses bactericidal activity, recent studies have used it to treat pathogenic infections in cutaneous and soft tissues, including dermatitis and chronic ulcerative wounds (Mai-Prochnow et al., 2014; Brehmer et al., 2015). Our recent study demonstrated that CAPJ with appropriate parameters has potent anti-microbial activity that is mediated through cell wall destruction and DNA breaks of the bacteria (Lou et al., 2019). Furthermore, the application of CAPJ to wounds created on rats showed a marked reduction in bacterial load compared to that of wounds not treated with CAPJ, indicating that CAPJ is effective when applied *in vivo*.

In addition to bactericidal effects, plasma-generated RONS possess wound healing activity, which may stimulate signaling pathways to regulate tissue repair in the skin (Isbary et al., 2010; Kubinova et al., 2017). Notably, RONS play a pivotal role in tissue repair, including cell migration and proliferation, as well as the formation of blood vessels at wound sites (Dunnill et al., 2017). Furthermore, RONS-induced endothelial cell proliferation after plasma treatment is related to the release of fibroblast growth factors (Ngo et al., 2014). Despite *in vitro* studies that have revealed valuable information on the role of plasma at the cellular level, few investigations have explored the detailed mechanistic and biological effects of plasma on skin wounds using live animal models.

Given the close association of plasma with antiseptic functioning, it has been found to be crucial for wound healing, although the specific molecules that contribute to this interaction still require further investigations. In the present study, we explored the effect of CAPJ into the plasma-activated medium (PAM) with different treatment durations under a pure He gas and particular composition of He and Ar mixed gases on human keratinocytes. We further identified its mechanism of action in several signaling pathways. Moreover, we assessed the healing activity of CAPJ when treating skin injuries using rat models. The results of the combined cell-based and animal studies demonstrate that the He and Ar mixed gases generated CAPJ can be developed as an effective treatment for cutaneous wounds.

## Materials and Methods

### Antibodies and Reagents

Antibodies specific against E-cadherin, N-cadherin, ERK, and phosphorylated ERK were purchased from Cell Signaling (Danvers, MA, United States). Antibodies specific to cyclin D_1_ and Cdk2 were purchased from Proteintech (Chicago, IL, United States). Antibodies against β-actin and Ki-67, rabbit or mouse horseradish peroxidase (HRP)-conjugated antibodies were purchased from Santa Cruz Biotechnology (Santa Cruz, CA, United States). All other reagents were purchased from Sigma-Aldrich (St. Louis, MO, United States).

### Cell Culture

Human keratinocytes (HaCaT cells, ATCC 12191) were cultured in Dulbecco’s modified Eagle’s medium (DMEM, Hyclone, Logan, UT, United States) containing 100 U/mL penicillin, and 100 μg/mL streptomycin. De-complement fetal bovine serum (10%; HyClone) was added to the culture medium. The cells were maintained at 37°C in a humidified atmosphere containing 5% CO_2_.

### Generation of CAPJ

All experiments with the plasma treatment were operated by the CAPJ as described in our previous study (Lou et al., 2019). An indirect treatment method was applied for the cell culture experiments, which the DMEM was activated by CAPJ before usage. Parameters of the CAPJ included a working voltage of 6.5 kV and a distance of 15 mm between CAPJ and DMEM surface. Five different treatment periods: 15, 30, 45, 60, and 90 s, were applied into DMEM under two different gas flowing conditions, one is the pure He gas under a flow rate of 5 slm (standard liter per min) (defined as He-CAPJ hereafter), and the other is the gas mixture of He and Ar with the flow rates of 5 and 0.5 slm (defined as He/Ar-CAPJ hereafter), respectively. The flow rate ratio of He and Ar gas mixture was 10:1 due to its ability for producing higher amounts of OH and NO radicals (Lou et al., 2019). The DMEM surface temperature was maintained at 34.5°C during CAPJ treatment as described in our previous reports (Lou et al., 2019).

### Cell Proliferation Assay

The MTT [3-(4,5-dimethylthiazol-2-yl)-2,5-diphenyltetrazolium bromide] assay (Sigma-Aldrich) was used to determine the effects of plasma gas on cell viability. Complete cell culture medium was pretreated with He-CAPJ and He/Ar-CAPJ for 15, 30, 45, 60, and 90 s, respectively, referring as PAM, followed by the incubation with HaCaT cells for an additional 24 h. MTT reagent was added to the cells and incubated for 4 h at 37°C. DMSO (Dimethyl sulfoxide) was added to each well to effectively dissolve the formazan crystals. Cell proliferation was measured by analyzing the ability of viable cells to reduce MTT reagent to formazan at the wavelength of 570 nm (Chen et al., 2017). The results were expressed as the means of three independent experiments performed in duplicate.

### Cell Migration Assay

HaCaT cells were culture in 12-well plates at a density of approximately 1 × 10^5^ and were grown to reach 100% confluence for 24 h. Two perpendicular scratches were performed by using a sterile p200 pipette tip. The culture media were replaced with PAM and cultured for an additional 24 h. Microscopic images of the wounded area were taken immediately after wounding at 24 h. The images were measured for the fields of each well by using ImageJ (National Institute of Health, Bethesda, MD, United States) (Chen et al., 2018). The average of three widths taken from upper, middle, and bottom of each wound area was analyzed for the closure scratch width (%) calculation using $\frac{W_{\text{i}}-W_{\text{f}}}{W_{\text{i}}}\times 100$, where W_i_ and W_f_ are initial and final wound widths, respectively (Romaldini et al., 2018). Each analysis was executed in duplicates and was repeated three times.

### Western Blot Assay

HaCaT cells (3 × 10^5^) were plated in 6-cm dishes and incubated with complete cell culture media which were pretreated with various conditions of He/Ar-CAPJ for 24 h. The treated cells were lysed with 100 μL RIPA reagent containing with protease and phosphatase inhibitors (Roch, Indianapolis, IN, United States). The cell lysates resolved by 10% SDS-PAGE and then performed the Western blot assay. The samples were transferred onto a polyvinylidene difluoride (PVDF) membrane (Millipore, Billerica, MA, United States) and immersed with 5% dehydrated skim milk to block non-specific protein binding. The membranes were incubated with primary antibodies as indicated in each experiment. The blots were then probed with the HRP-conjugated secondary antibody. The proteins of interest were detected using ECL Western blotting detection reagents (GE Healthcare, Piscataway, NJ, United States) and visualized by using Azure c400 system and AzureSpot Analysis Software (Azure Biosystems, Dublin, CA, United States). The signal intensity of each protein was quantified with UN-SCAN-IT software (Silk Scientific Corporation, Orem, UT, United States) as described previously (Lin et al., 2019). These data were expressed as the mean ± standard deviation determined from three independent experiments.

### Animal Study

**FIGURE 1:**
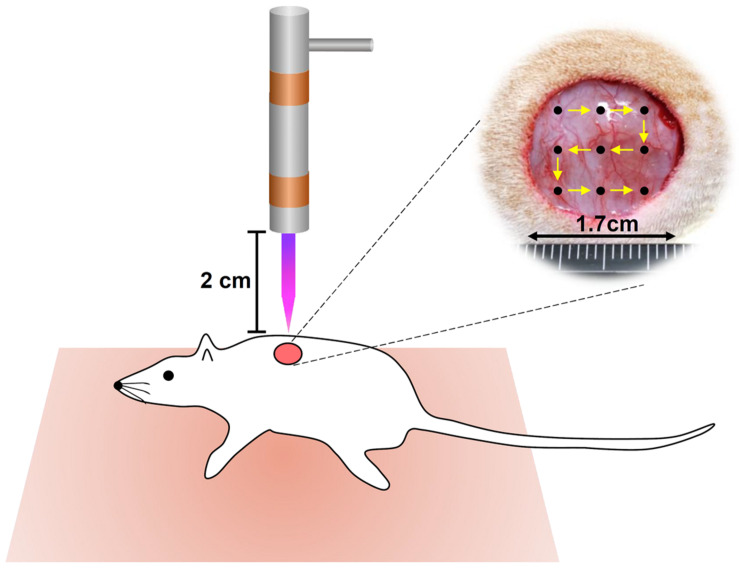
Schematic representation of CAPJ treated wounds in rats. Two full-thickness wounds, 17 mm in diameter, were created on both sides of a rat’s shoulder. Right: treated with He-CAPJ and He/Ar-CAPJ, left: untreated control (not shown).

The evaluation of CAPJ treatment was performed on cutaneous wounded male Sprague-Dawley (SD) rats. A total of twenty seven male SD rats (8 weeks old, BW 250 ± 30 g) were obtained from the National Laboratory Animal Center of Taiwan. The rates were treated in accordance with the Animal Care and Use Guidelines for Chang Gung University under a protocol approved by the Institutional Animal Care Use Committee (Approval No. CGU105-032). The experimental protocol was performed from July 1, 2018 to June 31, 2019, in accordance with the guidelines. Twenty one rats were separated into He-CAPJ (*n* = 6) and He/Ar-CAPJ (*n* = 15, amongst, 9 for histological analysis) groups. The working voltage, sample distance, and treatment period for the He-CAPJ and He/Ar-CAPJ treatments were 7.5 kV, 20 mm, and 60 s, respectively. Two full-thickness wounds with 17 mm in diameter were produced on both sides of a rat’s shoulders under anesthesia and muscle relaxant with zoletil (25 mg/kg) and rompun (10 mg/kg), respectively. The right-side wounds were treated with CAPJ once, and the left side wounds were kept untreated as controls for comparison. The CAPJ treatment on each wound was divided into 9 points starting from upper left corner to bottom right corner as shown in Figure 1 for evenly treated wound areas. Rats (*n* = 3) were euthanized by CO_2_ on days 3, 7, and 14 after He/Ar-CAPJ treatment. The tissue samples were collected from the both sides of cutaneous wounded rats for the followed histological analysis. The wounds and adjacent skin were removed by operating scissors with a diameter of 15 mm. The removed tissues were placed on tissue embedding cassettes and soaked in 10% neutral buffered formalin for tissue fixation and hematoxylin-eosin (H&E) staining, which were performed by a manufacturer (Helix Technology Co., Ltd., Taiwan). The evaluation of wound closure and tissue inflammation was executed based on histopathological examination and verified by a dermatological physician. Six rats were further used to evaluate the effect of CAPJ treatment period, which were treated for 3 min (right side) and 5 min (left side) under He/Ar-CAPJ with constant working voltage of 7.5 kV and sample distance of 20 mm. The photos of the wound areas were taken every other day until fully recovery and analyzed using ImageJ (National Institute of Health, Bethesda, MD, United States).

### Statistical Analysis

The relation of between-group comparisons was performed using the chi-square with Fisher exact test. Statistics analysis comparisons of more than two groups were evaluated using two-way analysis of variance (ANOVA). The statistical analysis was performed by using the SPSS program (version 18.0 for windows, SPSS Inc., Chicago, IL, United States). A *P*-value less than 0.05 was considered statistically significant.

## Results

### Establishment and Characterization of CAPJ

**FIGURE 2:**
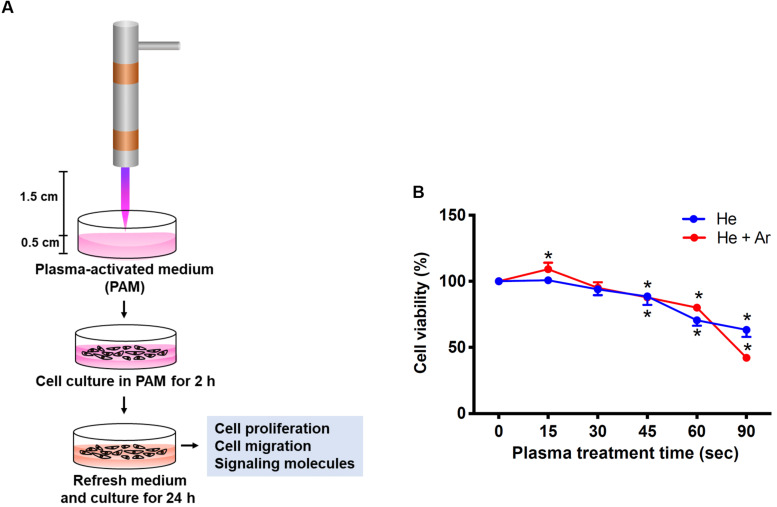
Experimental establishment of CAPJ system for the treatment of human keratinocytes. **(A)** The schematic configuration of the CAPJ device. CAPJ was operated with a working voltage of 6.5 kV. The gas flow rates of He and Ar were 5 and 0.5 slm, respectively. The distance between the CAPJ and DMEM surfaces was 15 mm. **(B)** HaCaT cells were exposed to plasma-activated medium (PAM) that was pretreated with either He-CAPJ or He/Ar-CAPJ for the indicated times. After culturing for 24 h, cell proliferation was analyzed using the MTT assay. These results were presented as the mean ± standard deviation from three independent experiments. Statistical significance was determined using the Student’s *t*-test (*P < 0.05).

The PAM was produced by the activation of the cell culture medium using CAPJ as shown in Figure 2A. The distance from the quartz tube nozzle to the liquid surface was fixed at 1.5 cm, and the working voltage was maintained at 6.5 kV. Various RONS generated in PAM by non-thermal plasma sources have been well reviewed in literature (Kaushik et al., 2018). Based on the optical emission spectroscopy (OES) spectra of He-based CAPJ, as observed in our previous study (Lou et al., 2019), the compositions and abundances of RONS generated by CAPJ are most likely dependent on the composition of the working gas. The intensity of the hydroxyl (OH) radical at a wavelength of 309 nm increased with increasing Ar gas flow rate but was unaffected at various application voltages (Lou et al., 2019). In addition, the intensity of nitrogen monoxide (NO) emission at a wavelength of 283 nm can be observed under the mixture of various Ar gas flow rates with fixed He gas, but not with pure He gas (Lou et al., 2019). These OH and NO radicals are the primary reactive species and relatively short-lived. These species are then converted to secondary and long-lived species (Kaushik et al., 2018). In this study, PAM was first pretreated with CAPJ under various conditions, such as different treatment durations and mixtures of the working gases (He or He/Ar). The major reactive species of NO_2_- concentrations in each PAM were carefully estimated using Criess reagents (Sigma-Aldrich, St. Louis, MO, United States). The concentrations of NO_2_ were gradually increased when the treatment time of PAM by He-CAPJ and He/Ar-CAPJ was increased (Supplementary Figure S1). The quantities of NO_2_- obtained in this study are consistent with that reported in literature (Kurake et al., 2016; Uchida et al., 2018). Human keratinocyte-derived cells, i.e., HaCaT cells, were then cultured with PAM for evaluation of cell proliferation, migration, and protein expression to extensively investigate the CAPJ interacting with cells (Figure 2A).

### He/Ar-CAPJ Enhances Keratinocyte Proliferation

HaCaT cells were exposed to PAM pretreated with He-CAPJ and He/Ar-CAPJ for different durations (15–90 s). After incubation for an additional 24 h, cell proliferation was analyzed using the MTT assay. As shown in Figure 2B, the keratinocyte proliferation is markedly increased when PAM was pretreated by He/Ar-CAPJ for 15 s. However, this trend was not observed in cells cultured with He-CAPJ treated PAM. In contrast, incubation of cells with He-CAPJ or He/Ar-CAPJ treated PAM for 45–90 s resulted in markedly inhibited cell proliferation in a time-dependent manner.

### He/Ar-CAPJ Promotes Keratinocyte Migration

**FIGURE 3:**
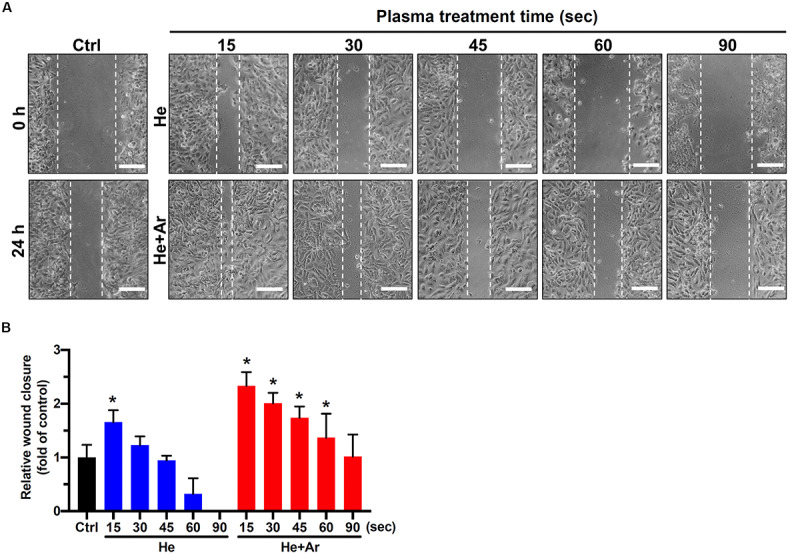
CAPJ facilitates human keratinocyte migration. HaCaT cells were treated with PAM that was pretreated with He-CAPJ or He/Ar-CAPJ for the indicated times. The cells were then subjected to a wound healing assay and cultured for 24 h to analyze cell migration activity. **(A)** The images were acquired after scratching for 0 or 24 h. Scale bars, 300 μm. **(B)** Cell migration activity was assessed by determining the relative wound closure, as described in section “Materials and Methods.” The data were presented as the mean ± standard deviation of three independent experiments. Statistical significance was determined using the Student’s *t*-test. *P < 0.05 when compared to the control group.

To further explore the impact of CAPJ on keratinocyte migration activity, a wound-healing assay was conducted. As shown in the cell images in Figure 3A and the relative wound closure results in Figure 3B, the migration activities of HaCaT cells were significantly elevated in PAM pretreated with He-CAPJ for 15 and 30 s but degraded for longer times from 45-90 s as compared to those of the untreated control groups (*P* < 0.05 for 15 s). Noticeably, the He/Ar-CAPJ pretreated group effectively promoted the cell migration as compared to the groups with untreated controls or He-CAPJ pretreatment. These results indicate that He/Ar-CAPJ treatment for shorter period possesses potent activity for promoting keratinocyte migration. According to the above cell proliferation and migration results, PAM pretreated with He/Ar-CAPJ for 15 s at a distance of 15 mm using a gas mixture of He and Ar with gas flow of 5.0 and 0.5 slm, respectively, was selected to perform the subsequent studies.

### He/Ar-CAPJ Activates Cell Proliferation Regulatory Molecules

**FIGURE 4:**
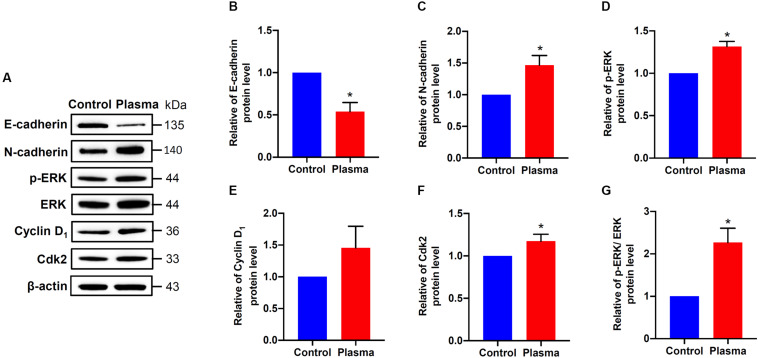
CAPJ regulates cell proliferation-related molecules in human keratinocytes. HaCaT cells were treated with PAM that was pretreated with He/Ar-CAPJ for 15 s, followed by incubation for 24 h. **(A)** Cell lysates were prepared to determine protein expression using western blot assay. The expression levels of **(B)** E-cadherin, **(C)** N-cadherin, **(D)** p-ERK, **(E)** cyclin D1, **(F)** Cdk2, and **(G)** ratio of p-ERK to ERK were determined by densitometric analysis and normalized to β-actin. Results are expressed as the mean ± standard deviation derived from three independent experiments. The cells untreated with PAM was set as 1.0. Fold change of each protein expressed in He/Ar-CAPJ treated cells was compared to PAM untreated control group. Statistical significance was determined using the Student’s *t*-test. *P < 0.05.

Since EMT and cell cycle progression were involved in wound healing (Das and Baker, 2016; Haensel and Dai, 2018), we therefore analyzed the effects of CAPJ on the regulators of EMT and cell cycle. HaCaT cells were exposed to He/Ar-CAPJ treated PAM followed by replacement of complete media and cultured for an additional 24 h, and the expression levels of E-cadherin, N-cadherin, cyclin D1, and Cdk2 were assessed using western blotting. As shown in Figure 4, markedly decreased E-cadherin (Figure 4B) and increased N-cadherin (Figure 4C) were observed in cells incubated with He/Ar-CAPJ treated PAM. Moreover, He/Ar-CAPJ effectively elevated the expression of p-ERK, a cell proliferation molecule (Figures 4D,G). The expression level of Ki-67 was increased upon cultured with PAM exposed to He/Ar-CAPJ (Supplementary Figure S2). We then determined the key effectors that contributed toward regulating the cell cycle. Our results showed that He/Ar-CAPJ significantly increased the expression levels of cyclin D1 and Cdk2 (Figures 4E,F). These results represent that He/Ar-CAPJ promotes keratinocyte proliferation and migration, which were evidenced by a decrease in E-cadherin with concomitant increases in N-cadherin, p-ERK, cyclin D1, and Cdk2.

### He/Ar-CAPJ Facilitates Wound Closure

**FIGURE 5:**
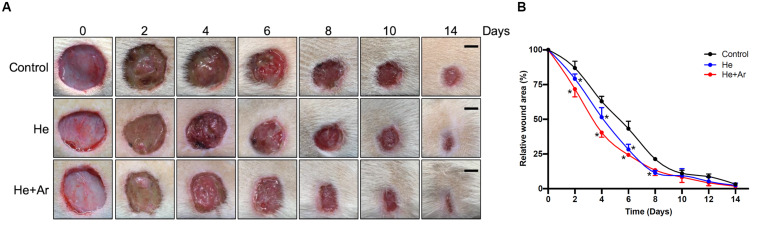
Images for skin wounds treated with CAPJ and enhanced cutaneous wound closure rate. Eighteen rats were separated into control (*n* = 6), treatment with He-CAPJ (*n* = 6) or He/Ar-CAPJ (*n* = 6) groups. **(A)** Photographs of cutaneous wound closures, which were untreated (control), treated with He-CAPJ and He/Ar-CAPJ on days 0, 2, 4, 6, 8, 10, and 14 are presented. Scale bars, 5 mm. **(B)** Wound closure rates were quantified on the indicated days. Statistical significance was determined using the Student’s *t*-test. *P < 0.05 when compared to control treatment.

**FIGURE 6:**
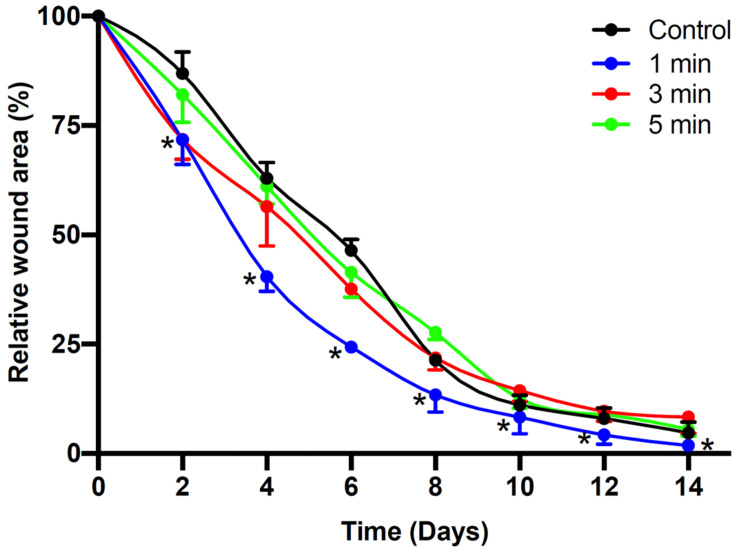
Time dependent CAPJ treatment on cutaneous wound closure rate. Cutaneous wounds were untreated (control) or treated with He/Ar-CAPJ for 1, 3, 5 min (*n* = 6 for each group). Wound closure rates on days 0, 2, 4, 6, 8, 10, 12, and 14 were quantified. Statistical significance was determined using the Student’s *t*-test. *P < 0.05 when compared to control treatment.

**FIGURE 7:**
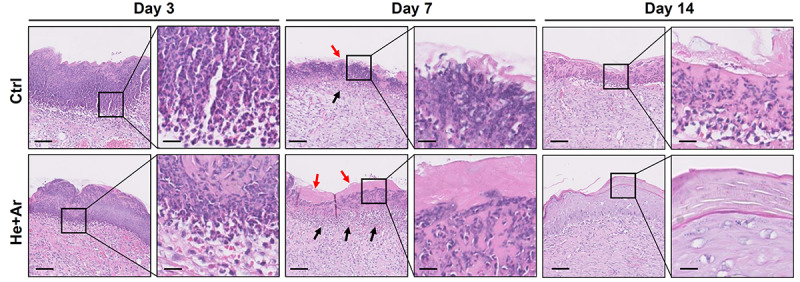
Histological analysis of wound healing with 60 s He/Ar-CAPJ treatment. Wound tissue sections were prepared on days 3, 7, and 14, and subjected to hematoxylin-eosin (HandE) staining. Black arrows indicate the formation of granulation tissues and red arrows show the epidermis. The magnified images are shown in the right panel of each cropped area. Scale bars in original and magnified images are 80 and 400 μm, respectively.

To further assess the effects of non-thermal plasma on wound closure, we established rat cutaneous wound models. The wound closure for each wound was measured on days 0, 2, 4, 6, 8, 10, and 14 after He-CAPJ and He/Ar-CAPJ treatment for 60 s. Our results showed that the wound area was significantly reduced by the plasma treatment as compared to that of the untreated control group shown in the photographs of the full thickness skin wounds and subsequent wound contraction (Figure 5A). Noticeably, a pronounced cutaneous wound contraction was occurred after treated with He/Ar-CAPJ for 8–14 days. Moreover, the effect of He/Ar-CAPJ on wound closure was better than He-CAPJ after treated for 2–8 days as depicted by the quantification of the wound area in Figure 5B. For the evaluation of plasma treatment period effect, wound closure was further determined by assessing the wound area treated with He/Ar-CAPJ for 1, 3, and 5 min on the indicated days. As shown in Figure 6, rat cutaneous wounds treated with He/Ar-CAPJ for 1 min featured a wound area that was substantially diminished as compared to those of control groups and other treatment periods (3 and 5 min). Skin tissue sections were further prepared for histological analysis. As shown in Figure 7, there was a decrease in inflammatory cell infiltration around the wounds that were treated with He/Ar-CAPJ as compared to those untreated control groups. In addition, the presence of granulation and re-epithelialization tissue were markedly increased in wounds treated with He/Ar-CAPJ after day 7 (framed region with magnified image in Figure 7). Together, these results illustrate that tissue contraction and re-epithelialization can promote wound closure after He/Ar-CAPJ treatment.

## Discussion

CAPJ, a cold or non-thermal plasma, has been reported to possess various biomedical applications *in vitro* and *in vivo* (Zimmermann et al., 2011; Arndt et al., 2013; Chatraie et al., 2018; Cheng et al., 2018; Gilmore et al., 2018; Guo et al., 2018). The CAPJ has been applied in the plasma medicine by direct and indirect approaches (von Woedtke et al., 2013). The indirect use of CAPJ for generating the PAM has been extensively studied for its potential in real application. Meanwhile, the direct use of CAPJ in the human or animal body has been adopted for evaluating the effects of wound healing and cancer treatment by plasma treatment. We conducted the indirect and direct CAPJ treatments and achieved remarkable results on the *in vitro* and *in vivo* studies, respectively.

In this study, we demonstrated that the He/Ar-CAPJ effectively enhanced keratinocyte proliferation and promoted wound closure with carefully controlled treatment duration. The most significant induction in cell proliferation was noted by He/Ar-CAPJ under a voltage of 6.5 kV and exposure for 15 s. Following the He/Ar-CAPJ treatment, we demonstrated signaling pathways that led to elevated levels of cell proliferation and cell cycle progression. The animal studies further showed that the He/Ar-CAPJ therapy to be responsible for accelerating cutaneous tissue repair in rat cutaneous wounds. In our current study, using cell-based and animal studies, we revealed the mechanism activated by the He/Ar-CAPJ treatment, namely, cell proliferation-regulating molecules, which in turn facilitated cell growth and wound healing.

The treatment effects of CAPJ for biomedical applications depend on key species formation and the abundance of RONS, which have rendered CAPJ treatment relevant to a broad range of research outcomes from cancer cell eradication to wound-healing promotion. In this study, based on the direct and indirect applications of CAPJ to SD rats and HaCaT cells, respectively, RONS and appropriate doses of CAPJ treatment can be adjusted by changing the CAPJ working gas composition and treatment durations (Lou et al., 2019).

NO, which was awarded as the Molecule of the Year in 1992 by the American Association for the Advancement of Science, is a specific molecule that acts as a biological messenger in the body, relaying information from the nerves to cells (Culotta and Koshland, 1992). Endogenous NO in terms of health acts as a functional cell regulator and messenger, and this activity is well-known. Notably, the amount of NO at low concentrations is beneficial, however, high levels of NO cause harmful effects on human health (Pacher et al., 2007). In addition, the production of NO and ROS functions as a potent defense against bacterial pathogens by decomposing macromolecules, including DNA and proteins (Hosseinzadeh Colagar et al., 2013). We previously reported that mixed He/Ar-generated CAPJ produces nitrogen and oxygen species, which leads to the destruction of the bacterial cell wall and damages double-stranded DNA to gain bactericidal activity (Lou et al., 2019). Decreasing bacterial loads of skin lesions attenuates inflammatory response and promotes tissue repair, thereby enhancing wound closure (Yu et al., 2011). Our present study showed that He/Ar-CAPJ with a proper cell treatment duration effectively activated cell proliferation, which in turn induced wound healing. These results combined with previous findings have elucidated the mechanism of the He/Ar-CAPJ is not only acting through antiseptic activity but also promoting tissue repair and in turn improving wound healing.

**FIGURE 8:**
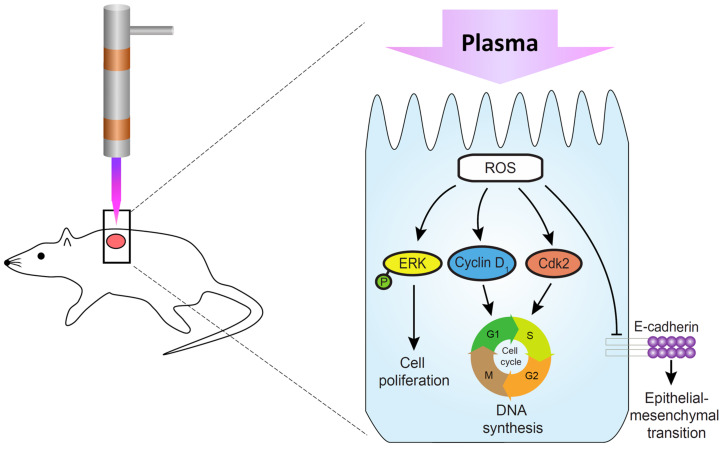
Molecular mechanism underlying the promotion of skin wound healing by CAPJ. He/Ar-CAPJ inhibits E-cadherin and activates cyclin D1, Cdk2, and p-ERK, which induce EMT and cell cycle progression, leading to cell proliferation and enhanced wound closure.

Our recent study has revealed that the He/Ar-CAPJ plasma can generate higher intensities of reactive oxygen species (ROS), including OH and NO radicals, nitrogen, and oxygen species (Lou et al., 2019). It has been reported that treatment of cells with low-temperature plasma can increase the production of ROS, activation of NF-κB, and protein expression cyclin D1, which promoted the cell cycle progression in responses to cell proliferation in wound healing (Liu et al., 2017; Shi et al., 2018). In addition, activation of ERK and p38 MAPK, which are responsible for the distinct activation of transcription factors NF-κB, can further enhance the skin wound closure in mice (Yun et al., 2014). In this study, we investigated the molecular mechanism through which the He/Ar-CAPJ induces keratinocyte migration, which is a key step in cutaneous wound healing (Usui et al., 2008). Our data showed that He/Ar-CAPJ with a proper time duration treatment elicits both the proliferation and migration of keratinocytes. These findings are supported by evidence that a reduction in the epithelial marker molecule, E-cadherin, leads to EMT activation (Gonzalez and Medici, 2014). Downregulation of E-cadherin enhances wound re-epithelialization (Terao et al., 2011), which is crucial for promoting epidermal cell migration and tissue repair (Haensel and Dai, 2018). The expression levels of cyclin D1 and Cdk2 are increased and drive the G1/S phase transition of the cell cycle, thereby enforcing cell proliferation (Lee et al., 2014). In addition, the activation of the ERK family, which is involved in the MAPK pathways, plays a pivotal role in the regulation of cell proliferation (Escuin-Ordinas et al., 2016). In consistent with our previous findings, our current results indicate that the He/Ar-CAPJ generates high intensities of reactive nitrogen and oxygen species for reducing E-cadherin and triggering p-ERK, cyclin D1, and Cdk2 expression, which are crucial factors for EMT and cell proliferation, resulting in the acceleration of cutaneous wound healing (Figure 8).

Since our animal model did not add the ring fixtures to restrict the surrounding of wound edge during the healing process (Figure 5), muscular contraction was occurred to enhance wound healing *in vivo*. In the treatment of CAPJ focusing in the central zone of the wound, the residual plasma gas may diffuse and influence on the margin of the wound edge, thereby inducing keratinocyte migration as well. In addition, the histological examination showed that keratinocyte migration could be activated to improve the wound healing by diminishing the wound size (Figure 7, day 14, He + Ar treatment group), which was consistently with previous studies (Lee et al., 2018; Zhang et al., 2019). Taken together, the *in vitro* study showing the keratinocyte activation can be histologically observed in the *in vivo* study and accompanying with muscular contraction that provide evidence for the underlying mechanisms of CAPJ to promote wound healing.

Full-thickness wounds destroy the dermis, which were healed by re-epithelialization and the formation of granulation tissue for filling the void of the wound before epithelial covering (Rittie, 2016). The re-epithelialization reaction then reaches its full significance, as a larger surface needs to be covered with new keratinocytes. In the present study, we used HaCaT cells as an *in vitro* assay platform, which is a long-lived, spontaneously immortalized human keratinocyte line (Carretero et al., 2008). Notably, HaCaT line exhibits basal cell properties and serves as a suitable model to follow the cell migration and release of inflammatory cytokines (Colombo et al., 2017). Our results found that plasma induction in HaCaT keratinocytes contributed to the signaling events and led to the activation of cell migration and proliferation. In addition to the *in vitro* data, we provided *in vivo* evidence, through plasma administration to excisional wounds in rates, showing improved wound healing by increasing the re-epithelialization and granulation tissue formation. Owing to the importance of keratinocyte migration substrates in re-epithelialization and muscular contraction, these findings combined the *in vitro* and *in vivo* studies provided more explicit mechanisms for the plasma that promoted the wound healing.

Our animal studies revealed that He/Ar-CAPJ treatment induces wound contraction on day 2, which is an initial step in the early phase of the cutaneous healing process (Reinke and Sorg, 2012). Further, there was a significant reduction in the wound area and obvious skin contraction in rats treated with He/Ar-CAPJ for 1 min as compared to that of the untreated control group on days 2–8. Histological analysis showed increased granulation tissue formation and re-epithelization, and mitigated inflammation after He/Ar-CAPJ treatment. These results are consistent with recent studies performed using plasma generated from Ar, He, and other gases (Shao et al., 2016; Kubinova et al., 2017; Schmidt et al., 2017).

## Conclusion

Our results demonstrate that the He/Ar-CAPJ with appropriate treatment duration enhanced skin tissue repair most likely by accelerating the cell cycle and activating cell proliferation pathways. The present study is the first to provide evidence that He/Ar-CAPJ generated reactive species possess bactericidal activity, increase granulation tissue formation, and alleviate inflammatory response, leading to enhanced cutaneous wound closure. Our observations revealed that the level of the ROS is very critical for wound healing, which is beneficial at low ROS concentrations yet harmful at high levels. This study provides insights into the mechanisms through which He/Ar-CAPJ enhances cell proliferation to repair skin lesions, suggesting the possibility of He/Ar-CAPJ therapy as a potent treatment for promoting wound healing.

## Data Availability Statement

The datasets generated for this study are available on request to the corresponding author.

## Ethics Statement

The animal study was reviewed and approved by the Institutional Animal Care Use Committee (Approval No. CGU105-032), Chang Gung University, Taiwan.

## Author Contributions

B-SL, J-HH, C-HL, and J-WL: conception or design of this work. C-MC, C-WH, H-YW, and P-YC: experimental study and data analysis and interpretation. B-SL, J-HH, C-HL, and J-WL: writing the manuscript. All authors contributed to the article and approved the submitted version.

## Conflict of Interest

The authors declare that the research was conducted in the absence of any commercial or financial relationships that could be construed as a potential conflict of interest.

## Supplementary Material

FIGURE S1 Concentrations of NO_2_^–^ in the PAM versus the treatment time of He-CAPJ and He/Ar-CAPJ. These data were measured using Criess reagents and expressed as the mean ± standard deviation determined from three independent experiments.

FIGURE S2 He/Ar-CAPJ increases Ki-67 expression in human keratinocytes. HaCaT cells were cultured with PAM treated with He/Ar-CAPJ for 15 s, followed by incubation for 24 h. Cell lysates were prepared to determine the protein expression levels of Ki-67 and caspase 3 by using western blot assay. β-actin expression was used as the loading control.

FIGURE S3 Original western blots for Figure 4A. Representative western blot results from three independent experiments are shown.
