# Supplementary figures and images for: Helium/Argon-Generated Cold Atmospheric Plasma Facilitates Cutaneous Wound Healing
Source: Front Bioeng Biotechnol. 2020 Jun 30;8:683. doi: 10.3389/fbioe.2020.00683 (PMC7338308; doi:10.3389/fbioe.2020.00683)

**Fig. S1**

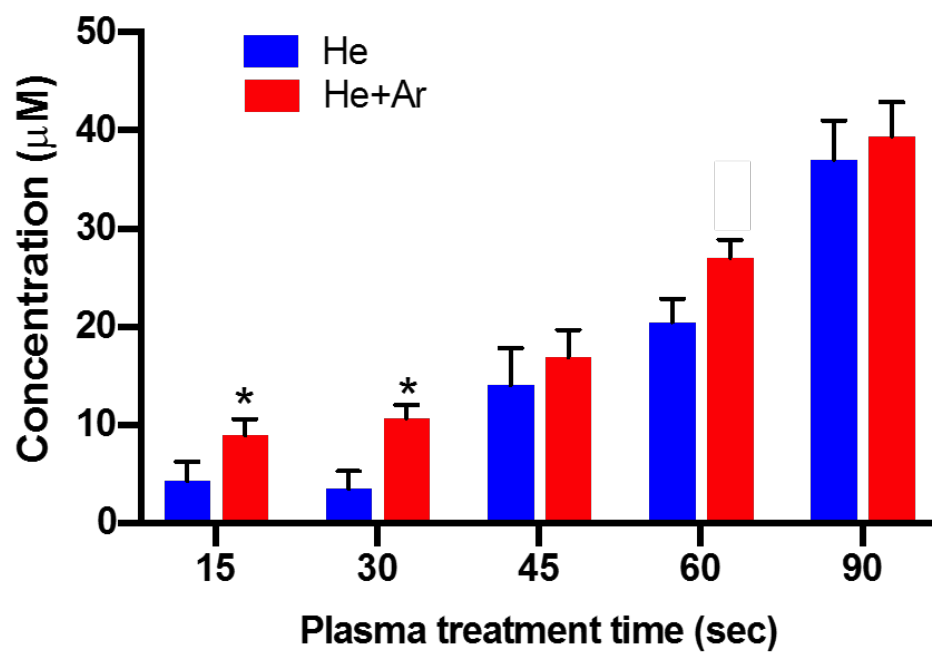

**Fig. S2**

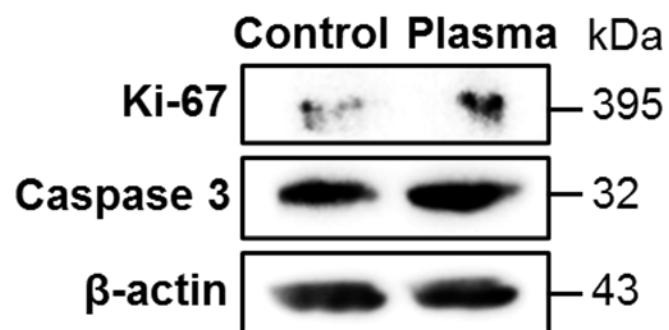

# Fig. S3

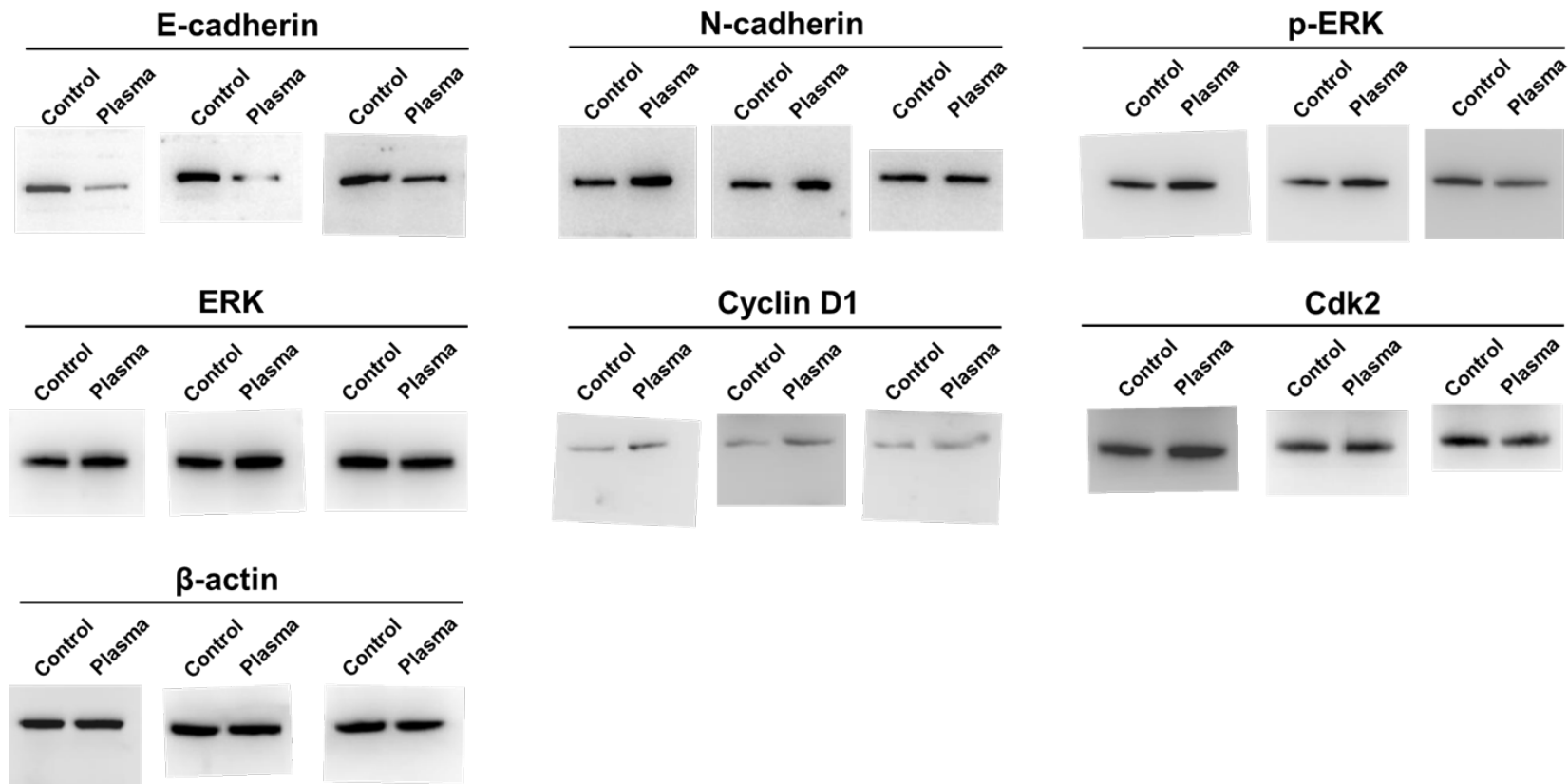

Supplement: FIGURE S1 — Concentrations of NO2– in the PAM versus the treatment time of He-CAPJ and He/Ar-CAPJ. These data were measured using Criess reagents and expressed as the mean ± standard deviation determined from three independent experiments. [file Image_1.pdf]
